# Recombinant Human Factor IX Produced from Transgenic Porcine Milk

**DOI:** 10.1155/2014/315375

**Published:** 2014-05-18

**Authors:** Meng-Hwan Lee, Yin-Shen Lin, Ching-Fu Tu, Chon-Ho Yen

**Affiliations:** Division of Animal Technology, Animal Technology Laboratories, Agricultural Technology Research Institute, No. 52 Kedong 2 Road, Zhunan Township, Miaoli County 350, Taiwan

## Abstract

Production of biopharmaceuticals from transgenic animal milk is a cost-effective method for highly complex proteins that cannot be efficiently produced using conventional systems such as microorganisms or animal cells. Yields of recombinant human factor IX (rhFIX) produced from transgenic porcine milk under the control of the bovine *α*-lactalbumin promoter reached 0.25 mg/mL. The rhFIX protein was purified from transgenic porcine milk using a three-column purification scheme after a precipitation step to remove casein. The purified protein had high specific activity and a low ratio of the active form (FIXa). The purified rhFIX had 11.9 *γ*-carboxyglutamic acid (Gla) residues/mol protein, which approached full occupancy of the 12 potential sites in the Gla domain. The rhFIX was shown to have a higher isoelectric point and lower sialic acid content than plasma-derived FIX (pdFIX). The rhFIX had the same *N*-glycosylation sites and phosphorylation sites as pdFIX, but had a higher specific activity. These results suggest that rhFIX produced from porcine milk is physiologically active and they support the use of transgenic animals as bioreactors for industrial scale production in milk.

## 1. Introduction

Hemophilia B (Christmas disease) is the second most common type of hemophilia and is caused by deficiency of factor IX (FIX), a vitamin K-dependent (VKD) serine protease zymogen (M*r* 57,000). FIX has a clearly defined four-domain structure that encompasses a *γ*-carboxyglutamic acid (Gla) rich-domain, two epidermal growth factor-like (EGF-like) domains, and a *C*-terminal serine protease domain [1]. Activation of FIX is achieved by the proteolytic cleavage of an activation peptide (M*r* 11,000) by either FXIa or FVIIa-tissue factor [2]. The protein then undergoes extensive posttranslational modification (PTM), including formation of seven disulfide bridges, addition of two *N*-glycans and six *O*-glycans, one phosphorylation, one sulfation, one hydroxylation, and up to twelve *γ*-carboxyglutamic acids [1]. Recombinant human FIX is currently produced from transgenic Chinese hamster ovary (CHO) cells and is commercially available as BeneFIX [3]. This is structurally and functionally similar to plasma-derived FIX (pdFIX), but with minor differences in posttranslational modification, such as sulfation, phosphorylation, and glycosylation [4, 5].

The feasibility of using transgenic animals as a source of recombinant proteins has been demonstrated for over two decades [6] and recombinant proteins have been produced and secreted in body fluids such as milk, blood, urine, and semen [7]. The mammary gland has been used successfully as a bioreactor to produce a variety of human proteins because milk is easily collected in large volumes and the gland is equipped to perform all of the necessary complex posttranslational modifications [8]. Both recombinant human antithrombin III (ATryn, GTC Biotherapeutics Inc.) expressed in goat milk and recombinant C1 esterase inhibitor (Ruconest, Pharming Group NV) expressed in rabbit milk have received marketing authorization [9, 10].

Bioactive rhFIX was first produced from transgenic sheep, but was only expressed at 25 ng/mL under the control of the mammary-specific *β*-lactoglobulin promoter [11]. The rhFIX produced from transgenic porcine milk was increased to 2~3 g/L and contained about 70–140 U/mL after introduction of a fused mouse whey acidic protein (WAP) promoter. However, only about 10% of the rhFIX was biologically active. Furthermore, high expression was only obtained during the early stage of the lactation period [12, 13]. In 1998, Wu developed the double-transgenic pig to produce both rhFIX and porcine lactoferrin from milk under the control of the bovine *α*-lactalbumin (*α*LA) promoter [14]. The expression of rhFIX in transgenic milk (tgFIX) reached 0.25 g/L during the entire lactation period. However, the activated form of FIX (FIXa), a major thrombogenic trigger, was also found in the transgenic porcine milk. The aim of this work was to purify and characterize the posttranslational modifications of tgFIX (rhFIXfrom transgenic porcine milk).

## 2. Materials and Methods

### 2.1. Collection of Transgenic Porcine Milk

Double-transgenic pigs carrying the human FIX [15] and porcine lactoferrin [16] genes driven by the bovine *α*-lactalbumin (*α*LA) promoter were obtained from the National Taiwan University, Taipei, Taiwan [17]. Oxytocin (20 IU) is given via sow ear vein injection through a winged infusion set (Meditop Corporation; SLG, Malaysia) to induce lactating from day 7 to day 30 after parturition. During milk collection, the sows have 8–10 piglets suckling concurrently and the sow requires stimulation from piglets to maintain the lactation capability. Two sows were provided about 900 mL milk in a collection procedure. The collected milk was defatted by centrifugation at 10,000 g for 20 min and stored at −80°C until purification.

### 2.2. Purification of tgFIX from Porcine Milk

The skim milk was mixed with 1/9 volume of 1 M sodium phosphate buffer (pH 5.8). The mixture was divided into aliquots, 50 mL Falcon Centrifuge Tube, and then frozen at −80°C until completely frozen. The frozen samples (200 mL) were completely thawed in a refrigerator at 4°C, followed by centrifugation at 10,000 g for 10 minutes to remove the precipitated caseins. The supernatant was diluted with a 1.5-fold volume of buffer A (50 mM Tris-HCl, pH 7.2) for subsequent purification by chromatography. The diluted fraction (409 mL) was applied to a Q-Sepharose column (2.6 cm × 15 cm). After the column was washed with 240 mL buffer A, the enzyme was eluted with 0.3 M NaCl in buffer A. The enzyme solution was then applied to a heparin-Sepharose column (1.6 cm × 15 cm), which was preequilibrated with buffer A containing 0.2 M NaCl. The active fractions were eluted with 0.3 M NaCl in buffer A. The pooled active fractions were applied to a Concanavalin A column (1.6 cm × 10 cm), which was preequilibrated with 40 mM Tris-HCl buffer, pH 7.2 containing 1 M NaCl, 2 mM MnCl_2_, and 2 mM CaCl_2_. The enzyme was eluted with 20 mM Tris-HCl buffer, pH 7.2 containing 0.5 M NaCl, and 0.2 M methyl *α*-D-mannopyranoside. The active fractions were pooled and stored at −20°C.

### 2.3. FIX Activity Assay

FIX activity was measured using the activated partial thromboplastin time (APTT) assay using the STA Compact analyzer (Diagnostica Stago, Inc., Asnieres, France) according to the manufacturer's protocol. Factor IX-deficient plasma was used as substrate. Factor IX activity was determined against a World Health Organization (WHO) International Standard in the range of 0.14 IU to 1.6 IU. The WHO International Standard (the 4th International Standard for FIX Concentrate) was purchased from The National Institute for Biological Standards and Control (NIBSC, Hertfordshire, UK). One unit of FIX activity was defined as the activity of FIX in 1 mL of pooled human plasma.

### 2.4. FIXa Activity Assay

Activated Factor IX (FIXa) activity was measured using a chromogenic assay (Hyphen BioMed, Neuville-Sur-Oise, France) according to the manufacturer's instructions using the manual end-point method.

### 2.5. Protein Concentration Determination

Protein concentration was determined using the Bradford method with bovine serum albumin as the standard.

### 2.6. Nonreducing SDS-PAGE and IEF

Electrophoresis was performed using a 10% nonreducing SDS-polyacrylamide gel electrophoresis system (Invitrogen, Carlsbad, CA). Isoelectric focusing (IEF) was performed using Invitrogen pH 3–7 gels according to the manufacturer's instructions (Invitrogen).

### 2.7. Analysis of Protein Posttranslational Modifications

#### 2.7.1. Oligosaccharide Determination

Oligosaccharides of tgFIX (1 mg) were released after overnight digestion at 37°C with PNGase F (1000 U/mL, Roche, Mannheim, Germany) in 20 mM ammonium bicarbonate buffer; pH 8.0. Oligosaccharides were analyzed on a CarboPac PA-100 column (25 cm × 4.6 mm, 5 *μ*m) using a Dionex HPAEC-PAD system (Dionex, Sunnyvale, CA). Bovine fetuin was used as a reference material.

#### 2.7.2. Monosaccharide Determination

The total carbohydrate content of tgFIX was determined by the orcinol-sulfuric acid method. Monosaccharide composition was analyzed by gas chromatography-mass spectrometry using a Hewlett-Packard model 6890 gas chromatograph, connected to a Hewlett-Packard 5973 mass-selective detector [18].

#### 2.7.3. Sialic Acid Determination

The sialic acid content released from tgFIX was determined using the Sialic Acid (NANA) Assay Kit (BioVision, Mountain View, CA).

#### 2.7.4. Gla Determination

*γ*-Carboxyglutamic acid (Gla) content was determined by amino acid analysis as described by Price [19]. One mg of protein was subjected to alkaline hydrolysis in 2 N KOH for 48 h at 110°C. Hydrolyzed amino acids were filtrated through a 0.2 *μ*m filter to remove precipitate. Supernatants were separated by high performance liquid chromatography on an AminoPac PA10 strong anion-exchange column with pulsed amperometric detector (Dionex, Sunnyvale, CA) using an elution buffer of 1 M sodium citrate. Purified *γ*-carboxyglutamic acid and aspartic acid (Sigma-Aldrich) were used as standards, and samples were compared with a control sample (pdFIX, Enzyme Research Labs, South Bend, IN). Determinations were performed in duplicate, and results are reported as an average.

#### 2.7.5. Protein Phosphorylation Determination

Protein phosphorylation was measured by the Phosphoprotein Phosphate Estimation Assay Kit (Pierce Biotechnology, Rockford, IL). The protein samples were incubated in 1 N NaOH at 65°C for 30 minutes to release phosphate. After neutralization with HCl, a mixture of malachite green and acidified ammonium molybdate was added to form a complex of phosphomolybdate and malachite green. The absorbance at 630 nm was read and the amount of phosphate was determined from a phosvitin standard curve.

#### 2.7.6. Protein Sulfation Determination

The tyrosine *O*-sulfate in tgFIX was determined by DABS-Cl method as described previously [20]. Activation peptide of tgFIX was digested with trypsin and purified by reverse phase HPLC [21]. Subsequently, 20 pmol/*μ*L activation peptide of tgFIX was digested with 0.02 *μ*g/*μ*L carboxypeptidase Y in 25 mM sodium citrate buffer, pH 6.0, at room temperature. Following digestion, 10 *μ*L mixture was added to 40 *μ*L 0.2 M sodium bicarbonate buffer at pH 9 and 100 *μ*L DABS-Cl (1.3 mg/mL in acetone) was added to produce DABS-amino acids. Reaction mixtures were incubated at 70°C for 10 min, dried in a vacuum, and redissolved in 70% ethanol for analysis using RP-HPLC.

### 2.8. Peptide Mapping

Transgenic FIX (157.5 *μ*g) was reduced with 2 mM dithiothreitol (Sigma) at 56°C for 1 h and alkylated for 1.5 h at room temperature (in the dark) with 5 mM iodoacetamide (Sigma). The sample was diluted with 50 mM ammonium bicarbonate and then 2 *μ*g of trypsin was added and incubated for 18 h at 37°C. After tryptic digestion, the sample was deglycosylated overnight at 37°C either with or without PNGase F. The peptide fragments were analyzed by HPLC (Waters, Milford, MA) with a reversed phase column (Biobasic-18, 5 *μ*m, 300 Å, 4.6 mm × 250 mm, Thermo Electron). The eluted fractions were collected manually according to the absorbance at 214 nm. Collected samples were dried by SpeedVac and then subjected to analysis by the CapLC System (Waters) using a capillary column (75 *μ*m i.d., 10 cm in length, MST, Taiwan) with a linear gradient of 30% to 80% acetonitrile containing 0.1% formic acid over 40 min. The separated peptides were online analyzed under positive survey scan mode on an ESI-Q-TOF (Micromass, Manchester, UK) instrument. All MS/MS spectra were processed using Mascot Distiller (Matrix Science, London, UK), and the resulting PKL files were searched using the Mascot search engine v2.2 (Matrix Science).

## 3. Results

### 3.1. Purification of tgFIX

**Figure 1:**
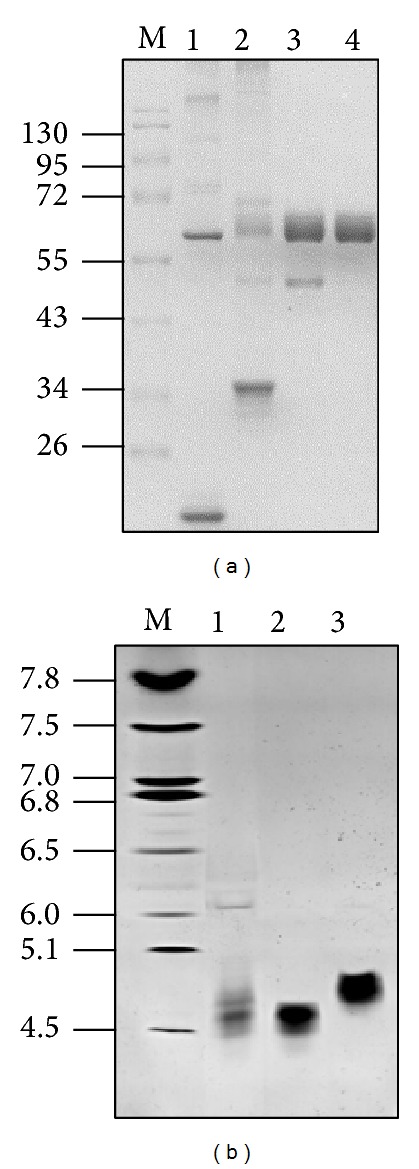
Electrophoretic analysis of rhFIX from transgenic porcine milk. (a) Nonreducing SDS-PAGE analysis. All lanes contain 10 *μ*g of protein. Lane M: molecular mass markers, Lane 1: whey. Lane 2: Q-Sepharose. Lane 3: heparin-Sepharose. Lane 4: Con A-Sepharose. (b) Isoelectric focusing (IEF) analysis. IEF was performed using Invitrogen pH 3–7 gels. Proteins were detected with Coomassie blue stain. Lane M: p*I* markers, Lane 1: plasma-derived FIX (pdFIX), Lane 2: FIX recombinantly produced from CHO cells (CHO-FIX), Lane 3: rhFIX purified from transgenic porcine milk (tgFIX).

The concentration of tgFIX in the raw milk of transgenic pigs was approximately 0.250 g/L. The results of the tgFIX purification from 450 mL skim milk are summarized in Table 1. The purified rhFIX had a specific activity of 293 U/mg. The ratio of FIXa/FIX was decreased to 1%. The final product appeared to be homogenous after analysis by nonreducing SDS-PAGE (Figure 1(a)). The *N*-terminal sequence was determined by Edman degradation method, and the sequence was found to be YNSGKLXXFVGG, which is identical to pdFIX.

### 3.2. Molecular Weight and Isoelectric Point

The apparent molecular mass of the purified enzyme was 57,000 Da as determined by nonreducing SDS-PAGE (Figure 1(a)). These results suggest that the enzyme is composed of a single polypeptide chain. The isoelectric point was estimated to be 4.8, which is higher than pdFIX and CHO-FIX (Figure 1(b)).

### 3.3. Monosaccharide and Sialic Acid Content

The total carbohydrate content of tgFIX was 7.86 ± 0.27% as determined by the orcinol-sulfuric acid method. The monosaccharide composition in tgFIX is given in Table 2. *N*-Glycolylneuraminic acid (NeuGc) was not found in the tgFIX oligosaccharide. The sialic acid content of FIX from three different sources was shown in Table 3. tgFIX contains 5.10 sialic acid in each molecule less than the one derived from plasma or from CHO cell. The difference of isoelectric point may be attributed to different sialic acid contents in these products (Table 3).

### 3.4. *N*-Linked Oligosaccharide

**Figure 2:**
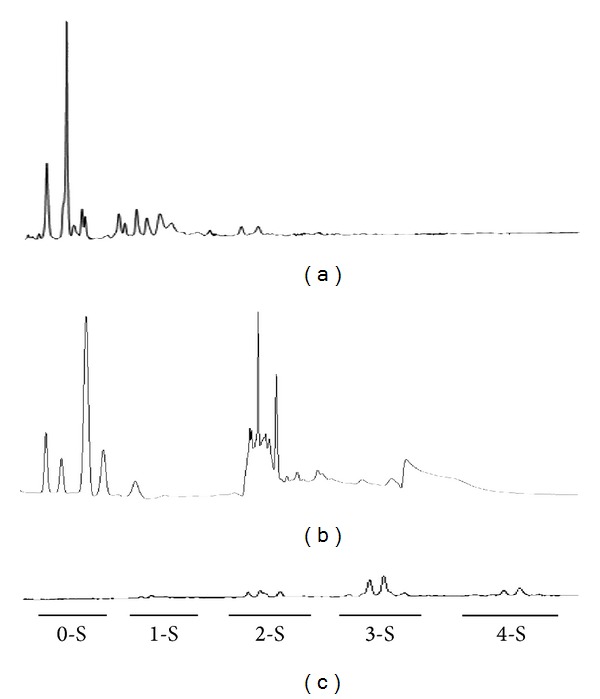
*N*-Glycans profile of (a) tgFIX, (b) pdFIX, and (c) bovine fetuin analyzed using HPAEC-PAD. Oligosaccharides were analyzed by the HPAEC-PAD system. The labels 0-S, 1-S, 2-S, 3-S, and 4-S indicate mono-, di-, tri-, and tetra-sialylated oligosaccharide groups, respectively.

In order to investigate the sialylation and complexity of glycans, the *N*-linked oligosaccharides from tgFIX were released with PNGase F and analyzed using the HPAEC-PAD system (Figure 2). Bovine fetuin, a well-characterized glycoprotein, was used as the standard in this study. Comparison of the *N*-glycan retention times indicated that the *N*-glycans derived from tgFIX contained neutral, monosialylated, and disialylated oligosaccharides (Figure 2(a)). In contrast, di-, tri-, and tetrasialylated glycans were predominant in pdFIX (Figure 2(b)).

### 3.5. Gla Content

The *N*-terminal Gla domain of FIX contains 12 potential sites for *γ*-carboxylated glutamic acid residues. In this study, tgFIX had 11.9 Gla residues/mol proteins, which was similar to the pdFIX that was analyzed as a control.

### 3.6. Protein Phosphorylation and Sulfation

A phosphorylated serine residue at position 158 of rhFIX has been shown to be associated with a lower* in vivo* recovery after infusion into hemophilia B patients [5]. Transgenic FIX had 0.36 phosphorus/mol proteins determined by Phosphoprotein Phosphate Estimation Kit. In contrast, pdFIX can contain up to 0.89 phosphorus/mol proteins.

The phosphorylation site on Ser158 of tgFIX was determined by digestion with trypsin and PNGase F; then the peptides were analyzed by MALDI-TOF MS. The MALDI-TOF MS spectrum showed two peaks with the observed* m/z* 3942.6 and 4022.6 that corresponded to the calculated MW of deglycosylation activation peptide of tgFIX without and with phosphorylation, respectively. The* m/z* 4022.6 peak was selected for MS/MS analysis and gained the amino acid sequence identical to deglycosylation activation peptide of tgFIX with a phosphorylation modified on Ser158 residue (data not shown).

The sulfation of Tyr residue on activation peptide was not detectable by using amino acid analysis by using DABS-Cl.

### 3.7. Peptide Mapping

**Figure 3:**
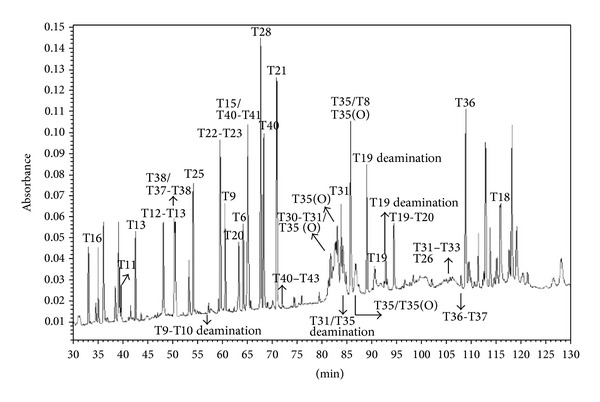
HPLC analysis of tgFIX digested by trypsin and PNGase F. Tn denotes the *n*th enzymatic peptide assigned from the *N*-terminal of tgFIX. For example, T1 indicates the first theoretical tryptic peptide from the protein *N*-terminal. Tn-Tn+1 or Tn-Tn+2 indicate peptides resulting from one or two missed cleavages, respectively. The collection time and matched peptide sequence are summarized in Table 4. Each peptide sequence was supported by MS/MS data and molecular weight match. A Mascot probability-based scoring algorithm was used to evaluate the identification.

For the determination of protein deamidation and oxidation, the tgFIX sample was treated by trypsin digestion in the presence or absence of PNGase F. The peptides were then subjected to reversed-phase HPLC separation. Each peak was collected manually and then analyzed by LC-MS/MS followed by a Mascot database search for peptide identification (Figure 3). The peptide identification details are summarized in Table 4. Some peptides were observed in adjacent HPLC fractions because of peak tailing and possible random error caused from manual collection. Overall, 80% sequence coverage of FIX was obtained. Only the T18 peptide was observed in a sample which had undergone trypsin plus PNGase F treatment. In addition, two deamidations were found in the T18 peptide. These data suggests that Asn157 and Asn167 were originally *N*-glycosylated. The tgFIX sample showed evidence of deamidation at Asn92, Asn199, and Asn347. Most of the T35 peptide fragments were found to be oxidatively modified at Met348. The oxidatively modified tgFIX still had high FIX coagulation activity, which suggests that Met348 does not play a key role in FIX coagulation activity.

## 4. Discussion

In our purification scheme, the tgFIX tightly bound to Q-Sepharose than most of whey proteins at pH 7.2, so that the anion exchanger can separate tgFIX from whey proteins. Heparin-Sepharose column is an affinity column specific binding to tgFIX that can remove the impurity to gain tgFIX in high purity. FIXa is the proteolytic product of FIX zymogen. The activation peptide with high glycosylation was released during the proteolytic process. Concanavalin A could specifically bind to glycoprotein. In the Concanavalin A column, the FIXa is easily removed to be only 1%. However, Concanavalin A is toxic to cell [22]. It is necessary to assay the residual concentration of Concanavalin A in the final product.

Factor IX is synthesized as a precursor polypeptide which requires proteolytic cleavage to remove propeptide for functional activity. In the Chinese hamster ovary (CHO) cells expression system, expression of factor IX, at high levels results in the secretion of a mixture of profactor IX and mature factor IX. In our experiment, the N-terminal sequence of purified tgFIX was found to be YNSGKLXXFVGG, which is identical to pdFIX. In addition, the proper propeptide processing was confirmed by peptide mapping. Our data suggested that the proFIX was removed during the purification scheme. However, the location of proFIX needs more experiments to be clarified.

Factor IX undergoes extensive PTM. The differences in PTM between pdFIX and CHO-FIX products have been extensively researched. Compared with pdFIX, CHO-FIX displays the absence of Ser158 phosphorylation (90% for pdFIX) [23]. The sulfation site of the activation peptide is largely filled (>90%) in pdFIX and largely unfilled in CHO-FIX (4-5%) [24]. The sulfation of Tyr residue on activation peptide of tgFIX was not detectable by amino acid analysis using DABS-Cl. The variability in sulfation and phosphorylation, in particular, is believed to impair* in vivo* recovery following intravenous infusions of CHO-FIX compared to pdFIX [23, 25].

Factor IX is a VKD protein that undergoes carboxylation to the fully *γ*-carboxylated mature zymogen, which is then secreted into the circulatory system. Plasma-derived FIX has twelve Gla residues in the *N*-terminal Gla domain. The Gla domain is responsible for Ca^2+^ binding, which is necessary for the binding of FIX to membrane phospholipids. Expression of recombinant proteins in mammalian cell culture in industry is suitable for the production of proteins with high PTMs; however, inefficient *γ*-carboxylation still remains a major problem with regard to VKD proteins [26]. Transgenic animal technology offers an effective expression system for high posttranslationally modified proteins. It was shown that the biologically active subpopulation of rhFIX was only about 10% in the milk of transgenic pigs that had high expression levels of 2~3 g/L under the control of the WAP promoter. The *γ*-carboxylation PTM is rate limiting for recombinant FIX at 0.2~0.3 g/L [13]. In this study, undercarboxylated (inactive) tgFIX protein was still expressed in mammary glands but the ratio was reduced to 25–30% under control of the bovine *α*-LA promoter (data not shown). The undercarboxylated rhFIX in milk could be separated from the fully carboxylated form by Q-Sepharose chromatography resulting in purified tgFIX with 11.9 Gla residues per rhFIX molecule.

FIX can be activated to be FIXa (M*r* 46000) by FXI and/or FVIIa/tissue factor complex in the presence of calcium and phospholipids [27]. Milk contains both calcium and phospholipid surfaces and a number of proteases that have the potential to degrade foreign proteins but their specific activity does not appear to be high enough to activate FIX to FIXa. In our study, FIXa is activated before the milk collection. The FIX activity was measured using the activated partial thromboplastin time (APTT) assay using the STA Compact analyzer. The high level of FIXa may interfere with the FIX assay. We also measured the FIXa activity by chromogenic assay in each purification scheme. The chromogenic assay is used to determine the activated FIX activity only.

Sialic acid in the terminal of both *N*-linked and *O*-linked glycans affects absorption, half-life, and clearance from the serum, as well as the physical, chemical, and immunogenic properties of glycoproteins [28]. Several biopharmaceuticals produced in transgenic animal milk contain a lower content of sialic acid. For example, recombinant human erythropoietin purified from the milk of transgenic pigs has a higher isoelectric point, higher hydrophobicity, and lower sialic acid content than the native protein [29]. *N*-linked glycans of antithrombin from transgenic goat milk [30] and lactoferrin from transgenic cow milk [31] contain less sialic acid than the corresponding glycans found in humans. In addition, tgFIX was shown to have a higher isoelectric point and lower sialic acid content in this study and in a study by Gil et al. [32]. The elimination half-life (*t*_1/2*β*_) of CHO cell-produced rhFIX determined by a conventional monoclonal sandwich ELISA assay was approximately 5.0 h [33] whereas the *t*_1/2*β*_ of tgFIX after a single intravenous administration was 9.34 h [34].

Two main types of sialyl residues are found in biopharmaceuticals produced by mammalian expression systems, for example, *N*-acetyl-neuraminic acid (NeuAc) and *N*-glycolyl-neuraminic acid (NeuGc). Anti-thrombin III produced by transgenic goats contains two types of sialic acid, NeuAc and NeuGc. Although NeuGc can be found in porcine proteins [32], the tgFIX produced in both this study and a study by Gil et al. [32] contained only NeuAc. NeuGc residues are undetectable in human plasma and are potentially immunogenic to humans. These data demonstrate that the pig is a better choice of animal than goat for the production of recombinant human proteins.

CHO cells have been successfully used for two decades with similar posttranslational modifications to their native counterparts. The major disadvantages of mammalian cell culture systems are their relatively low production levels and high cost [7]. The lack of availability and affordability of clotting factor concentrates remains a major disappointment to hemophiliacs in the recombinant era. The possible need for factor IX is about 2 kg per year. Houdebine estimated that four transgenic sows is enough supplied the annual demand of factor IX [7]. However, based on the lower expression level (0.35 g/L) and the yield of purification process (assumed to be 10%), the minimum herd size of 192 transgenic sows is required in ideal condition for worldwide market. Transgenic animals offer the latest promising alternatives to commercial bioreactors for recombinant protein production. This may open the door for future approval of hemophilia therapies derived from such technology to yield low cost recombinant clotting factors that can be used throughout the developing world.

## 5. Conclusion

In this study, the data clearly demonstrate the expression and characterization of recombinant human FIX by transgenic pigs. The present work demonstrates the potential use of transgenic pigs for the production of tgFIX for the treatment of hemophilia B patients.

## Figures and Tables

**Table 1:** Summary of a representative purification of tg-FIX from transgenic porcine milk.

Step	Volume (mL)	Total protein (mg)	Total activity (U)	Specific activity (U/mg)	Purification (fold)	Yield (%)	FIXa/FIX (%)
Whey	409	11342	34026	3	1	100	19
Q-Sepharose	100	211	25531	121	40	75	18
Heparin-Sepharose	23	42	10752	256	85	32	11
Concanavalin A-Sepharose	20	12	3516	293	98	10	1

**Table 2:** Monosaccharide content of tgFIX purified from transgenic porcine milk.

Monosaccharide	mol/mol tgFIX
*N*-Acetylgalactosamine	5.16
*N*-Acetylglucosamine	14.12
*N*-Acetylneuraminic acid	4.26
Fucose	1.25
Galactose	11.88
Glucose	1.41
Mannose	5.73
Xylose	4.32

**Table 3:** Sialic acid content of FIX.

Sialic acid	mol/mol FIX
tgFIX	5.10 ± 0.04
pdFIX	8.69 ± 0.12
CHO-FIX	6.77 ± 0.20

**Table 4:** Peptide matching of tgFIX digested by trypsin plus PNGase F. Fractions were collected manually from HPLC separation. This represents data from the same experiment as shown in Figure 3. The peptide identities suggested by LC-MS/MS analysis are listed. Those fractions in which no peptide was identified are removed from the list.

Fraction number	Time	Peptide number	Position	Observed *m*/*z*	Match peptides
1	35.1~35.8	T16	135–142	418.1808	VSVSQTSK
5	38.8~40.2	T11	95–100	871.442	CEQFCK
6	42.4~43.1	T13	107–116	615.7483	VVCSCTEGYR
7	48.0~48.8	T12-T13	101–116	620.5777	NSADNKVVCSCTEGYR
8	50.2~51.2	T38	395–400	744.471	YGIYTK
		T37-T38	393–400	465.2822	GKYGIYTK
10	54.0~54.8	T25	253–265	752.9462	IIPHHNYNAAINK
11	57.0~57.8	T9-T10	81–94	565.2617	NCELDVTCNIKNGR + Deamidated (NQ)
12	59.4~60.2	T22-T23	229–248	580.8007	ITVVAGEHNIEETEHTEQKR
13	60.3~61.2	T9	81–91	683.3281	NCELDVTCNIK
14	62.8~63.8	T20	202–214	698.3452	VDAFCGGSIVNEK
15	63.9~64.6	T6	38–43	406.2174	TTEFWK
16	64.8~65.8	T40-T41	404–411	540.3281	YVNWIKEK
		T15	123–134	688.851	SCEPAVPFPCGR
17	67.4~68.0	T28	302–312	586.8006	FGSGYVSGWGR
18	68.2~68.8	T40	404–409	411.7751	YVNWIK
19	70.4~71.4	T21	215–228	785.9102	WIVTAAHCVETGVK
20	71.6~72.6	T40–T43	404–415	761.9668	YVNWIKEKTKLT
21	80.8~81.4	T35	342–358	679.6353	FTIYNNMFCAGFHEGGR + Oxidation (M)
22	81.4~82.8	T30-T31	317–327	638.3962	GRSALVLQYLR
		T35	342–358	679.6486	FTIYNNMFCAGFHEGGR + Oxidation (M)
24	82.8~83.4	T35	342–358	679.6478	FTIYNNMFCAGFHEGGR + Oxidation (M)
25	83.4~84.0	T31	319–327	531.8453	SALVLQYLR
26	84.1~85.0	T31	319–327	531.8246	SALVLQYLR
		T35	342–358	674.6462	FTIYNNMFCAGFHEGGR + Deamidated (NQ)
		T34-T35	339–358	779.7193	STKFTIYNNMFCAGFHEGGR
27	85.4~86.2	T35	342–358	1010.9647	FTIYNNMFCAGFHEGGR
		T35	342–358	679.6509	FTIYNNMFCAGFHEGGR + Oxidation (M)
		T8	64–80	1062.4471	DDINSYECWCPFGFEGK
		T34-T35	339–358	589.0386	STKFTIYNNMFCAGFHEGGR + Oxidation (M)
28	86.4~87.6	T35	342–358	1010.9542	FTIYNNMFCAGFHEGGR
		T35	342–358	679.6524	FTIYNNMFCAGFHEGGR + Oxidation (M)
29	88.6~89.6	T19	181–201	742.7371	VVGGEDAKPGQFPWQVVLNGK + Deamidated (NQ)
30	90.2~91.0	T19	181–201	742.3987	VVGGEDAKPGQFPWQVVLNGK
31	92.2~93.2	T19	181–201	742.7443	VVGGEDAKPGQFPWQVVLNGK + Deamidated (NQ)
34	94.2~95.2	T19-T20	181–214	901.2115	VVGGEDAKPGQFPWQVVLNGKVDAFCGGSIVNEK
37	106.0~107.0	T31–T33	319–338	781.7831	SALVLQYLRVPLVDRATCLR
		T26	266–293	1076.8988	YNHDIALLELDEPLVLNSYVTPICIADK
38	107.4~108.2	T36-T37	359–394	957.4534	DSCQGDSGGPHVTEVEGTSFLTGIISWGEECAMKGK
39	108.4~109.2	T36	359–392	1214.5544	DSCQGDSGGPHVTEVEGTSFLTGIISWGEECAMK
43	115.6~116.6	T18	146–180	1313.9320	AEAVFPDVDYVNSTEAETILDNITQSTQSFNDFTR + 2 Deamidated (NQ)
